# Evaluation of JATSdecoder as an automated text extraction tool for statistical results in scientific reports

**DOI:** 10.1038/s41598-021-98782-3

**Published:** 2021-09-30

**Authors:** Ingmar Böschen

**Affiliations:** Institute of Psychology, Psychological Methods and Statistics, University Hamburg, Von-Melle-Park 5, 20146 Hamburg, Germany

**Keywords:** Psychology, Medical research

## Abstract

The extraction of statistical results in scientific reports is beneficial for checking studies on plausibility and reliability. The R package *JATSdecoder* supports the application of text mining approaches to scientific reports. Its function *get.stats()* extracts all reported statistical results from text and recomputes *p* values for most standard test results. The output can be reduced to results with checkable or computable *p* values only. In this article, *get.stats()*’s ability to extract, recompute and check statistical results is compared to that of *statcheck*, which is an already established tool. A manually coded data set, containing the number of statistically significant results in 49 articles, serves as an initial indicator for *get.stats()*’s and *statcheck*’s differing detection rates for statistical results. Further 13,531 PDF files by 10 mayor psychological journals, 18,744 XML documents by *Frontiers of Psychology* and 23,730 articles related to psychological research and published by *PLoS One* are scanned for statistical results with both algorithms. *get.stats()* almost replicates the manually extracted number of significant results in 49 PDF articles. *get.stats()* outperforms the *statcheck* functions in identifying statistical results in every included journal and input format. Furthermore, the raw results extracted by *get.stats()* increase *statcheck*’s detection rate. *JATSdecoder*’s function *get.stats()* is a highly general and reliable tool to extract statistical results from text. It copes with a wide range of textual representations of statistical standard results and recomputes *p* values for two- and one-sided tests. It facilitates manual and automated checks on consistency and completeness of the reported results within a manuscript.

## Introduction

The technical revolution goes along with a steady increase in the total number of yearly published scientific articles. Computers have become incredibly fast and enable us to deal with huge amounts of textual data, which has never been easier to preselect, access, store and process before. The PubMedCentral database^1^ alone stores more than 3 million open access documents related to the biology and health sciences.

Along with the publication boom, several scientists have expressed their doubts about the robustness of many scientific results carried out^2–6^. The Open Science Collaboration^7^ tried to replicate 100 experiments with a psychological background and could only replicate between 23 and 63% of the original findings, depending on the subject and definition of a successful replication. This result led to the so-called replication or reproducibility crisis in psychology.

Besides the many problems arising with overly small sample sizes^2,6,8^, psychological research is often based on ‘WEIRD’ selective samples^9^ and standardized statistical test procedures like uninformed nil-null-hypothesis testing^3,10^ with an *α*-error probability of .05. Uninformed nil-null-hypothesis testing refers to statistical procedures that are applied on empirical data to test null-hypotheses of no correlation, zero difference or no effect with an undirected test.

A crucial, paradoxical difference between theory testing in physics and psychology is emphasized by Meehl^3^:

‘In the physical sciences, the usual result of an improvement in experimental design, instrumentation, or numerical mass of data, is to increase the difficulty of the ‘observational hurdle’ which the physical theory of interest must successfully surmount; whereas, in psychology and some of the allied behavior sciences, the usual effect of such improvement in experimental precision is to provide an easier hurdle for the theory to surmount.’

Using nil-null-hypothesis tests, researchers that seek for significant results can apply several questionable research practices like optional stopping, multiple testing or postdiction^11,12^ to increase the possibility of a false positive result.

Journal editors and readers usually seek for new, sensational results rather than replications reporting differing or supporting results to the original findings. Also, many researchers face a scoring system that values the pure quantity and impact of published articles more than the reliability and robustness of their findings. This surrounding may lead to a lot of flawed results and endangers scientific credibility. John et al.^12^ surveyed over 2,000 psychologists about their involvement in questionable research practices and found that the percentage of respondents who committed to have engaged in at least one questionable practice was surprisingly high (up to 78% in a self report).

Within the highlighted world, an incredibly large amount of research findings is obviously contaminated with many spurious findings and errors. Still, corrections are published rather rarely and errors are preserved in the literature. Therefore, it can be quite beneficial for scientists, authors, reviewers, editors and/or search engines to summarize a scientific report in terms of its text parts and main study characteristics.

There are several tools and techniques to check specific statistical results on plausibility. The GRIM test^13^ is an easily performed calculation to check a mean of a Likert scale on plausibility, if the given sample size is known. In their analysis, Brown et al.^13^ found 36 out of 71 articles (51%) that reported at least one inconsistent mean. An automated tool to check *p* values in reports of various statistical test results is *statcheck*^14^ which is described in detail later. Nuijten et al.^15^ found that half of all published psychology papers that use null-hypothesis significance testing contained at least one *p* value that is inconsistent with its test statistic and degrees of freedom.

Besides the practical use of such checking procedures, implausible results should not be considered as a proof for a statistical error or a corrupt report and always be analyzed case by case. On the other hand, a result that passes a plausibility check does not directly imply an adequate, objective or even correct contextual decision, especially in terms of causality and generalizability. No algorithm can replace an informed expert, evaluating the whole study design, sampling methods, sample characteristics, operationalization and adequateness of the statistical procedure applied, to decide if the conclusions made are plausible, correct, or even valid. Still, an automated extraction of the reported statistical results is the key element to perform a check on completeness and plausibility quickly. An automated identification of studies that use certain statistical methods/measures or sample sizes can be very helpful for selection processes in meta-analyses and systematical reviews.

Previously to a detailed description and comparison of the algorithms by an evaluation on varying input formats, a terminology for distinct representations of statistical results is introduced.

### Terminology for destinct representations of statistical results reported in text

Generally, any letter or letter-number combination pointing to a numeric value with an operator (<, >, $=$, $\le$, $\ge$) is here considered to be a potential statistical result. A statistical result can be a descriptive measure as well as a test result. Statistical test results mostly consist of a varying set of results (test statistic, degree/s of freedom, an effect measure, *p* value, confidence interval and/or a Bayes Factor). There are many widely used statistical tests. Results that contain a *Z*-, *t*-, *F*-, $\chi ^2$, *r*-, *H*-, *Q*-, $G^2$, *U*-statistic or Bayes Factor and/or a measure of effect ($\beta$, Cohen’s *d*, $\eta ^2$, *OR*, *RR*, $R^2$) are defined as statistical standard results here.

Although there are guidelines about how to report statistical results (e.g. APA style), they are not reported in this standardized manner consequently (e.g. *p* value only). It makes sense to differentiate between different reporting practices of test results in terms of the level of their completeness and post processability. A statistical test result that enables a recomputation of an also reported *p* value is defined as checkable here (e.g.: ‘$t(89) = 1.96, p = .05$’). Test results that enable a computation of a non-reported *p* value (e.g.: ‘$t(89) = 1.96$’) will be called computable. A checkable result is always computable. The third set of test results is reported in a manner, in which no recomputation of a reported or unreported *p* value is possible (e.g.: ‘$r = .12, p<.05$’, or ‘$t>2$’). These results will be called uncomputable here.

### The R Package *JATSdecoder*

The R package *JATSdecoder*^16^ supports the application of text mining approaches to scientific reports by processing XML documents that are structured with the Journal Archiving Tag System NISO-JATS^17^. The NISO-JATS is an HTML tag standard to store scientific articles without any layout parameters. Graphical content is hyper referenced.

*JATSdecoder*’s functions make use of simple and sophisticated text extraction and manipulating algorithms that can cope with a wide range of textual and technical representations of content in NISO-JATS coded documents. The built-in function *JATSdecoder()* extracts a set of metadata (title, author, publishing dates etc.), the abstract, sectioned text and reference list. The structured output is very useful for individual searches and extraction procedures, as it facilitates these tasks on individually defined text parts (e.g. section titles, method section, reference list) and metadata.

*JATSdecoder*’s function *study.character()* performs multiple text selection and manipulation tasks on the list created by *JATSdecoder()* and extracts key study characteristics like number of reported studies, the statistical methods, software and correction procedures for multiple testing used. Its function *get.stats()* outputs all detected statistical results including descriptive measures (mean, sd, CI, Cronbach’s alpha) as a vector which is then further processed. Detected *Z*-, *t*-, *F*-, $\chi ^2$, *r*-, *H*-, *Q*-,$G^2$, *U*-statistics or Bayes Factors and corresponding effect measures $R^2$, $\beta$, *OR*, *d* and/or $\eta ^2$ are formatted into a data frame with numerical values and operators stored in separated columns.

To increase *get.stats()*’s detection rate for computable and checkable results, users can activate its arguments ‘*T2t*’ and/or ‘*R2r*’. Statistics denoted with capital letter T or R respectively will then be treated as *t*- or *r*-values, which may not be appropriate. Activating its argument ‘*estimateZ*’ makes *get.stats()* estimate *Z*-statistics for beta- and d-values reported with standard error but no test statistic.

If possible, a recomputed *p* value for an undirected null hypothesis is added. If desired, also *p* values for directed tests can be outputted if a computation is possible (only *t*-, *Z*-, *r*-values). The resulting data frame can be reduced to computable results only (recalculation of *p* value is possible, e.g. ‘$r(18) = .12$’), checkable results (recomputable result with *p* value), or outputted with all detected standard results (e.g.: no *p* value check possible: ‘$r = .12, p = .61$’ or *p* value only).

Deviations in reported and recomputed *p* values may be multicausal (directed test, rounding, typo, extraction or compilation error). Therefore, a check for completeness and plausibility of the results is not done automatically. Users checking a manuscript should always manually countercheck the extracted results and inconsistencies.

A non-computed, although expected to be computed *p* value, a non-reported but computed *p* value, or a completely missed out result in the output of detected standard results may be an indicator for an incompletely reported result within the text. Warning messages are returned if *p*-, *r*-, or $R^2$-values are reported that are outside their valid range.

Statistical results reported in tables are explicitly not captured by *get.stats()*, as the compilation of tables cannot be performed reliably. Here *statcheck* differs from *study.character()*, as it always analyses the whole textual content of an HTML or PDF to text converted file and captures test results from tables, if they are reported in a full textual manner and not with named columns, which is much more frequently done in practice.

To extract the statistical methods mentioned in an article, *study.character()* tries to split the NISO-JATS document into four sections (introduction, method, results, discussion). Its function *get.method()* performs a heuristic driven feature extraction process, to output the statistical methods listed in the method and results section. It finds the specification of a method, that contains the descriptive term in front of a set of search terms, which most commonly used statistical procedures have in common (e.g.: test, regression, anova, method, theorem, interval, algorithm, etc.). Users can enlarge the result space by defining additive search words in its argument ‘*add*’. The current heuristic enables an extraction of new, still unknown statistical procedures, if they are named with one of the already specified or user adjustable search terms at the end (e.g. ‘*JATSdecoder* algorithm’). Methods with a specifier behind the search term (e.g. ‘test on homogeneity of variances’) cannot be identified.

To identify the total number of studies reported in a document, the software and correction method used, fine-tuned dictionary searches are performed on preselected text parts and phrases. Software identification can be enhanced by adding further software search patterns.

Despite its wide extraction capabilities, the focus here is solely on *study.character()*’s function *get.stats()* and its ability to extract and post-process statistical results out of NISO-JATS formatted research articles. A simple web interface to extract and check statistical results within single articles in different formats (PDF, XML, HTML, DOCX) is hosted at: www.get-stats.app.

Several conversion tools that transform PDF documents into a post processable text object exist. One sophisticated converter is the Content ExtRactor and MINEr (CERMINE)^18^ which extracts metadata, full text and parsed references from a PDF file and makes it storable in different formats (plain text, NISO-JATS XML, etc.). The implementations of most steps are based on supervised and unsupervised machine learning techniques, which simplifies the procedure of adapting the system to new document layouts and styles^18^.

Language and type setting features allow very individual ways of expressing one and the same bit of information. This is especially relevant when processing text with many formulas, indices, special characters (operators, Greek letters, hyphens, separators, brackets, etc.) and synonymously used characters (Greek/Latin small letter beta: $\beta$ , sharp german s: ß, HTML beta: $` \& beta;'$). In electronic documents characters can be represented by different character codecs (UTF-8, ASCII, Unicode, hexadecimal, HTML, etc., or even pictures) which generally makes each extraction and compilation task on numerical results and other content more complicated.

When compiling PDFs with *CERMINE*, a wide range of compilation errors can occur (e.g.: missed operators, handling of subscripts, undetected Greek and special letters). *JATSdecoder*’s function *letter.convert()* unifies many letter representations and corrects most PDF and *CERMINE* specific conversion errors. This enables *JATSdecoder* to also reliably process PDF files that were converted to NISO-JATS coded XML files by *CERMINE*.

*JATSdecoder*’s algorithms have been developed iteratively based on the PubMedCentral article collection and about 10,000 PDF files from different journals, that were converted with CERMINE. *get.stat()* is designed for numbers that are reported with a dot as a decimal separator.

### How *get.stats()* works

A two-step process is performed to extract the reported results within a text and recalculate the reported *p* value with *get.stats()*. First, the input text is converted into sentences, squared into round brackets. Only those sentences are selected, that contain at least one letter and an operator followed by a number. To extract the reported test statistic, degrees of freedom, corresponding effect measure and *p* value, they are split at a set of words (e.g. ‘and‘, ‘or‘, ‘were‘, ‘of‘, etc.) and at words followed by a comma. If multiple test results are identified in a text snippet (e.g. more than one t- or *p* value), it is further split up, assuming a test statistic is reported in front of its *p* value. Text that appears in front and behind the results is removed with regular expressions (e.g. the text behind the last reported operator pointing to a number). The first result is a vector with unified representations of sticked results, starting with any letter or letter-number combination with, if present, degrees of freedom in round brackets, pointing to a number with an operator. Several heuristics to unify the representation of overly big and small numbers are applied. Before extracting the actual value of each standard result and the reported *p* value, regular expressions are used to remove labels of test statistics. Every targeted standard result is extracted from the sticked results with an individual heuristic that copes with a variety of reporting styles. The recognized value of the test statistic, its operator, the degrees of freedom and *p* value of each sticked result is returned as a cell in a matrix, which represents the second output. Each type of result is stored in a separated column, which greatly facilitates further post-processing and identification tasks. In standard mode, the recalculation of *p* values is performed based on the result matrix using basic R functions for distribution functions (‘$2*(1-pnorm(Z)$)’, ‘$1-pchisq(chi2, df)$’, etc.). Users can activate an additional recomputation for one-sided *t*- and *Z*-tests, as well as *r*-values that are reported with degrees of freedom.

### The R package *statcheck*

The R package *statcheck*^14^ performs an automated detection of statistical test results reported in APA style. It is capable of extracting adequately reported *Z*-, *t*-, *F*-, *r*-, *Q*- and $\chi ^2$-statistics with adequately reported degrees of freedom and a *p* value to check the result on plausibility (see:^19^). *statcheck* recomputes the corresponding *p* value and flags inconsistencies to the reported *p* value. The built-in functions work on plain text (*statcheck()*), HTML (*checkHTML()*) and PDF files (*checkPDF()*).

Nuijten et al.^20^ validated *statcheck* on the manually coded analysis of errors in all reports of statistically significant *t*-, *F*- and $\chi ^2$-test results in 48 articles, published by the Journal of Personality and Social Psychology and Journal of Experimental Psychology: Learning, Memory, and Cognition. *statcheck* extracted 648 out of 1,120 results (57.9%) in the comparative dataset (one retracted study with 28 significant results, that was part of the original analysis, was excluded by the *statcheck* authors).

Screening 39,717 articles published by eight journals with *statcheck* Nuijten et al.^15^ found checkable results in 16,695 documents (42%). Here *statcheck* flagged 8,273 (49.6 %) of these 16,695 articles with at least one inconsistency. Hartgerink^21^ analyzed 167,318 articles published by APA, Springer, Sage, and Taylor & Francis with *statcheck* and found 688,112 checkable statistical results in 50,845 articles (30.4%).

As noted by Schmidt^22^ *statcheck*’s identification rate for statistical results is rather low. This is in part due to its inability to handle statistical results that are not reported exactly according to APA style, reported with degrees of freedom (or label) in subscript, that contain semicolons instead of commas, square brackets instead of parentheses, effect sizes in-between test statistic and *p* value^23^.

As there is growing enforcement not to rely on the standard *p* value thresholds of ‘$p = .05$’ too much but rather change it to ‘$p<0.005$’^24^, report effect sizes and confidence intervals instead^25^, or even turn away from frequentist methods entirely^26^, *statcheck* will ever get worse in doing a good job as a detector of statistical results in text, the more these demands are implemented in practice.

As *statcheck* falsely flags inconsistencies in *p* values, when appropriate correction methods have been applied (*p* value correction for multiple testing instead of $\alpha$-error adjustment) and therefore might encourage users not to use the appropriate methods, Schmidt^22^ concludes that *statcheck* is an unsuitable software to detect errors in statistical results and should rather not be used.

**Table 1:** Some examples of statistical results and the extracted standard results by get.stats() with its argument ‘T2t = TRUE’ and statcheck().

Type	Example	*get.stats()*	*statcheck()*
APA t-test result	‘t(12) = 1.9, $p>.05$’	‘t(12) = 1.9, $p>.05$’	‘t(12) = 1.9, $p>.05$’
APA F-test result	‘F(2, 12) = 3.12, $p<.05$’	‘F(2, 12) = 3.12, $p<.05$’	‘F(2, 12) = 3.12, $p<.05$’
APA r-test result	‘r(13) = .52, $p<.05$’	‘r(13) = .52, $p<.05$’	‘r(13) = .52, $p<.05$’
APA Z statistic in front of line	‘Z = 1.9, $p>.05$’	‘Z = 1.9, $p>.05$’	
APA Z statistic behind white space	‘Z = 1.9, $p>.05$’	‘Z = 1.9, $p>.05$’	‘Z = 1.9, $p>$.05’
APA Q-test result	‘Q(13) = .52, $p>$.05’	‘Q(13) = .52, $p>$.05’	‘chi2(13) = .52, $p>$.05’
Non APA t-test result	‘t = 1.9, df = 12, $p>$.05’	‘t(12) = 1.9, $p>$.05’	
Non APA F-test result	‘F = 3.12, df1 = 3, df2 = 14, $p<$.05’	‘F(3, 14) = 3.12, $p<$.05’	
Semicolon as separator	‘F(1, 46) = 21; $p<$.05’	‘F(1, 46) = 21, $p<$.05’	
High df with comma	‘F(12; 1,222) = .12, $p<$.05’	‘F(12, 1222) = .12, $p<$.05’	
High F result with semicolon as separator	‘F(12; 122) = 2,123; $p<$.05’	‘F(12, 122) = 2123, $p<$.05’	
Test result with ns instead of *p* value	‘t(12) = 1.9, ns’	‘t(12) = 1.9’	‘t(12) = 1.9, ns’
APA t-test result with effect size	‘t(12) = 1.9, d = .2, $p>$.05’	‘t(12) = 1.9, d = .2, $p>$.05’	
Multiple completely reported results	‘all ts(27)>4.2, $p<$0.01’	‘t(27)>4.2, $p<$0.01’	
Multiple incompletely reported results	‘all rs<0.2, all ps>.01’	‘r<0.2, $p>$.01’	
Only *p* value	‘$p<$0.05’	‘$p<$0.05’	
t statistic with numbered index	‘t2(122) = 1, $p>$.05’	‘t(122) = 1, $p>.05$’	
F statistic with lettered index	‘Finteraction(1, 46) = 2.8, $p<$.05’	‘F(1, 46) = 2.8, $p<$.05’	
$\hbox {G}^{2}$ goodness of fit statistic	‘G2(41) = 2.3, $p<$.05’	‘G2(41) = 2.3, $p<$.05’	‘chi2(41) = 2.3, $p<$.05’
Result with capital T instead of t	‘T(12) = 2.33, $p<$.05’	‘t(12) = 2.33, $p<$.05’	‘t(12) = 2.33, $p<$.05’
Result with fraction	‘t(12) = 1/2, $p>.05$’	‘t(12) = .5, $p>.05$’	
Result with corrected *p* value	‘t(122) = 3, $p<$.05/2’	‘t(122) = 3, $p<$.025’	
Two reported statistics in a row	‘r(12) = .22, Z = .75, $p = .45$’	‘r(12) = .22, Z = .75, $p = .45$’	
Incomplete but p computable result	‘chi2(12) = 12.3’	‘chi2(12) = 12.3’	
Test on beta without z-/t value	‘beta = 22, SE = .77, $p<$0.01’	‘beta = 22, SE = .77, $p<$0.01’	
Test on beta without z-/t- nor *p* value	‘beta = 1.1, SE = .71’	‘beta = 1.1, SE = .71’	
Delta $R^2$ result	‘$\Delta$R2 = 34%, $p<$.05’	‘R2 = .34, $p<$.05’	
BayesFactor result with beta and *p* value	‘beta = 1.2, BF(10)<1, $p = .72$’	‘beta = 1.2, BF(10)<1, $p = .72$’	
BayesFactor result with H0:H1	‘BF(01) = 2e2’	‘BF(10) = 0.005’	
Pearson correlation	‘rp(12) = .22, $p = .45$’	‘$p = .45$’	‘r(12) = .22, $p = .45$’
Pearson correlation	‘sr(12) = .22, $p = .45$’	‘$p = .45$’	‘r(12) = .22, $p = .45$’
Pearson correlation	‘pr(12) = .22, $p = .45$’	‘$p = .45$’	‘r(12) = .22, $p = .45$’
Other statistic: LR statistic	‘LR(12) = .1, $p>.05$’	‘$p>.05$’	‘r(12) = .1, $p>.05$’
Other statistic: $\hbox {I}^2$ statistic	‘$\hbox {I}^2$(22) = 1, $p>.05$’	‘$p>.05$’	‘chi2(22) = 1, $p>.05$’
Any hight 2 statistic	‘$^2$(22) = 1, $p>.05$’	‘$p>.05$’	‘chi2(22) = 1, $p>.05$’
A statistic	‘A(12) = 2.3, $p<$.05’	‘$p<$.05’	‘chi2(12) = 2.3, $p<$.05’
B statistic	‘B(12) = 2.3, $p<$.05’	‘$p<$.05’	
c statistic	‘c(12) = 2.3, $p<$.05’	‘$p<$.05’	‘chi2(12) = 2.3, $p<$.05’
d statistic	‘d(12) = 2.3, $p<$.05’	‘$p<$.05’	‘chi2(12) = 2.3, $p<$.05’
Interval result	‘.12<r<.22, $.87<p<.65$’	‘r = .22’	

Representations are presented in an easy readable format instead of the resulting data tables extracted. Empty cells represent no detections.

### Distinguishing features of *get.stats()* and *statcheck*

Compared to *statcheck*, that looks out for a narrow set of exact pattern matches in a string, *get.stats()* deals with almost any result reported in text. In contrast to *statcheck* commas as well as semicolons used as separators can be handled by *get.stats()*.

Before extracting the actual value of every detected standard result, *get.stats()* selects, splits and cleans up all sentences presenting statistical results. *get.stats()* extracts and post-processes many standard results that are labeled or indexed. It performs several transformations of the textual representation of numbers in text. Fractions, as well as results reported with a ‘e⌃number’ or a percent sign are compiled to decimal numbers, commas in large numbers ($\ge 1000$) are removed. The output should therefore not be treated as an exact representation of the reported results.

Whereas *statcheck*’s functions always analyze the full document or text entered, *study.character()*’s argument ‘*text.mode*’ enables an extraction with *get.stats()* on specific text parts (1: full text and abstract, 2: method and result section/s, 3: result section/s only).

*statcheck* treats non-significant *p* values reported with ‘ns’ as checkable results, whereas *get.stats()* treats such results as computable, if the reported result allows a recomputation of the *p* value (e.g.: ‘$t(18) = 1.1, ns$’).

Table 1 lists some potential results of a vector with identified sticked results by *get.stats(x,output = ‘stats’)*. The selected examples demonstrate how *get.stats()* and *statcheck()* differ, in terms of their ability to detect, extract and check statistical results reported in text.

In most of the listed examples, *get.stats()* extracts all contained standard results defined earlier, whereas *statcheck()* fails to detect many of the results at all and extracts some results inadequately. Any squared statistic, as well as any statistic denoted with one of 18 upper- or lowercase letters (except: B, F, N, R, T, Q, W, Z) that is reported with its degrees of freedom in brackets is interpreted as $\chi ^2$-test results by *statcheck*. ‘rp’-, ‘sr’-, ‘pr’- and ‘LR’-statistics are interpreted as correlations by *statcheck()*, which, in part, may be correct. *get.stats()* does not classify these letter combinations as standard results. Results reported as intervals may cause missing or erroneous detections by *get.stats()* as the last example in Table 1 demonstrates.

## Method

To evaluate and compare the *JATSdecoder* and *statcheck* algorithms in terms of their practical precision and reliability in extracting statistical results in prespecified text parts, two analyses are performed with different input formats.

First, the total number of manually extracted statistically significant *t*-, *F*- and $\chi ^2$-statistics in the method and result section of 49 articles by Wicherts et al.^27^ is compared to the number of computable, statistically significant *t*-, *F*- and $\chi ^2$-results extracted from the method and result section with *study.character(x,text.mode = 2)* and *statcheck*’s algorithms. The differences between the manually coded data and *study.character()*’s detections are described case by case.

The vector containing the extracted sticked results by *get.stats()*, as well as an index/label removed version are further processed with *statscheck*’s function *statcheck()* to demonstrate how the letter correction in *CERMINE* converted PDF documents increases *statcheck*’s detection rate for checkable test results.

All non- or incorrectly converted but corrected operators, that are replaced with ‘$< =>$’ by *letter.convert()* are converted to ‘=’ before being processed with *statcheck()*. Labels and/or indices of reported test statistics are removed with simple regular expressions. As no other $\alpha$-error level was identified in the 49 studies, all results that lead to a recomputed *p* value $\le .05$ or that are reported with ‘$p\le .05$’ are selected to compare the number of extracted significant results. Next the same article collection is analyzed by each algorithm with no limitations on *p* values nor type of statistics nor on the part of text. The distribution of the number of detected results is displayed in box plots for each procedure and input format.

The second analysis demonstrates that *get.stats()*’s high performance and detection rate for statistical results also holds for much bigger article collections. An unrestricted search for statistical standard results is performed on 13,531 converted PDF articles, published between 2010 and 2020 in 10 mayor journals of psychology (J. of Abnormal Psychology, J. of Beh. Neuroscience, Psychophysiology, J.o. Child Psychology, Depression & Anxiety, J. of Management, Psychology & Aging, Psychological Medicine, J. of Family Psychology, Personality and Social Psychology Bulletin). A further 18,744 raw NISO-JATS coded XML documents, published by the open access journal *Frontiers in Psychology* and 23,730 ‘research-article’ tagged documents with the pattern ‘*[Pp]sych*’ in its *keyword*- or *subject*-tag published by *PLoS One*, serve for the analysis.

As no manually coded data exists for this big data set with varying input formats, the number of identified standard results by *get.stats()* (‘all’, ‘computable’ and ‘checkable’) is compared to that detected by *statcheck*’s functions with global descriptive measures. The total and relative amount of articles with detectable results and the total sum of detected results is presented for every journal and algorithm setting, as well as some descriptive measures for articles with identifiable results (mean, sd, median, IQR, .99 quantile, maximum, processing time).

All converted PDF documents are passed to *get.stats()* and *checkHTML()* as they contain HTML standard coding. The native PDF files are processed with *checkPDF()* and the preprocessed vector with sticked results extracted by *get.stats(x,output="stats")* is passed to *statcheck()*. Non-significant *p* values reported with ‘ns’ are excluded before counting *statcheck*’s detections to enable a comparison of the extracted number of checkable results. As the PMC bulk download contains native XML files only, no processing with *checkPDF()* is performed for these studies.

### Data, input formats, PDF conversion software, hardware

Native PDF and browser (Mozilla Firefox 80) generated HTML files serve for the first analysis of 49 empirical research articles analyzed in Wicherts et al.^27^. 13,531 PDF files that were published between 2010 and 2020 in 10 mayor journals of psychology were downloaded manually with the library license owned by University of Hamburg. Letters to the editor and corrections are not part of this article collection. PMC’s bulk download ftp-server (ftp://ftp.ncbi.nlm.nih.gov/pub/pmc/oa_bulk/) was used on 01.01.2021 to download all available native NISO-JATS coded XML documents, published by *Frontiers in Psychology* (18,744 XML files) and *PLoS One* (143,615 XML files).

The open source software *CERMINE*^18^ was used to convert each PDF file into a NISO-JATS coded XML, before being processed with *JATSdecoder*’s function* study.character()* or *get.stats()*.

All extractions and analyses were performed with an AMD@Epyc 7452 32-core processor running with Linux Ubuntu 20.04.1 LTS and the open source software R 3.6^28^. To enable multicore processing, the R package *future.apply* was used^29^.

## Results

### Evaluation of *get.stats()* detection rate with manually coded data and *statcheck*’s functions

**Figure 1:**
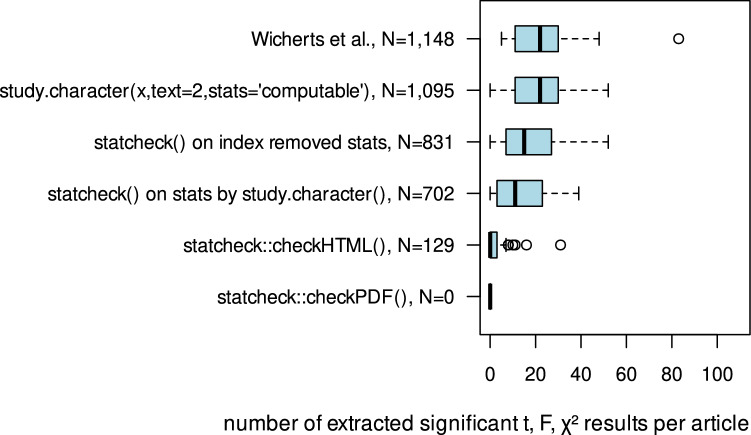
Total sums of extracted significant *t*-, *F*- and $\chi ^2$-test results per method and distributions of number of extracted significant *t*-, *F*-, $\chi ^2$-test results per article and method.

First the total number of significant *t*-, *F*- and $\chi ^2$-test results that was extracted manually by Wicherts et al.^27^ is compared to the number of significant results extracted by *study.character()* and *statcheck*’s functions. Figure 1 displays the distribution of identified significant *t*-, *F*-, and $\chi ^2$-statistics per paper for the applied extraction method and input format.

*study.character()* identifies 1,095 significant results in the method and result sections compared to 1,148 results extracted by Wicherts et al.^27^. *checkHTML()* only detects 129 significant *t*-, *F*- and $\chi ^2$-test results within the full text of the browser generated HTML documents. *checkPDF()* could not extract a single statistic out of the same raw material, that was converted with *CERMINE* to become processable with *JATSdecoder*. The extracted sticked results extracted by *get.stats()* within the method and result section/s and an additional removal of labels/indices by simple regular expressions, enhances *stacheck()*’s ability to detect and check results (702 and 831 results respectively) and supersedes *checkHTML()*’s functionality for browser generated HTML files.

**Table 2:** Causes for deviations in the number of extracted statistically significant *t*-, *F*-, and $\chi ^2$-test results within method and result section/s per paper by Wicherts et al.^27^ and study.character().

ID	N results Wicherts	N study.character()	$\Delta$	multiple result	Error in *p* value	p operator	Result in footnote	Other section	Fit index	Tabled result	CERM-INE	Un-clear
2	13	15	2			2						
3	33	35	2					2				
6	30	49	19	8		5						6
9	9	10	1		1							
10	21	22	1									1
13	21	22	1				1					
17	37	51	14			13						1
18	11	14	3	3								
19	39	40	1			1						
21	46	52	6				6					
23	35	39	4				4					
26	33	37	4					4				
28	21	22	1	1								
29	24	26	2				2					
32	16	20	4						3			1
33	23	27	4			2						2
34	24	26	2				1					1
36	29	30	1			1						
39	20	21	1			1						
42	28	31	3					3				
43	27	29	2			2						
44	8	20	12	1			3					8
45	7	11	4		1	1						2
46	9	12	3		2	1						
49	30	37	7				7					
5	83	33	$-50$							$-48$		$-2$
16	32	23	$-9$								$-9$	
22	20	7	$-13$							$-13$		
24	30	29	$-1$					$-1$				
31	6	0	$-6$							$-6$		
35	5	3	$-2$									$-2$
38	15	0	$-15$							$-15$		
41	48	33	$-15$							$-16$		1
47	36	27	$-9$							$-11$		2
48	45	8	$-37$							$-37$		
Sum			$-53$	13	4	29	24	8	3	$-146$	$-9$	21

Here *study.character()* extracts 53 significant results less, than were found in the manual analysis. As 146 significant results are reported in tables and not extracted by *study.character()*, 93 additive significant results are identified. Table 2 summarizes each of the 35 cases with deviations to Wicherts et al.^27^. There are several reasons for a higher detection rate by *study.character()*. 13 checkable test results that are reported for several tests (e.g. ‘all ts(18)>3, ps<.05’) are extracted by *study.character()*. Four results that are incorrectly reported with ‘$p>.05$’, although they are significant, were not included by Wicherts et al.^27^ but found with *study.character()*. As none of the 49 *CERMINE* converted PDF files contains readable operators, *letter.convert()* inserts "$<=>$" to these empty or badly captured text parts. An insignificant result reported with ‘$p>.05$’ is therefore indistinguishable from a significant result reported with ‘$p<.05$’. This leads to 29 false positive inclusions in total. 24 results that are reported in footnotes and identified by *study.character()* seem not to be included in the original analysis. One result reported in the description of an experiment seems to be included in the original analysis but is not identified by *study.character()*, as only method and results sections are selected. In three articles, *study.character()* detects a total of nine results in the method sections that seem not to be included in the manual extraction. Three goodness of fit $\chi ^2$-statistics are excluded by Wicherts et al.^27^ and included by *study.character()*. Nine significant result are missed by *study.character()* because some text parts or section titles got lost while PDF conversion. Compared to the manually coded data, there are four missed results and 25 detections by *study.character()* that cannot be explained and might be due to bad captures by Wicherts et al.^27^.

**Figure 2:**
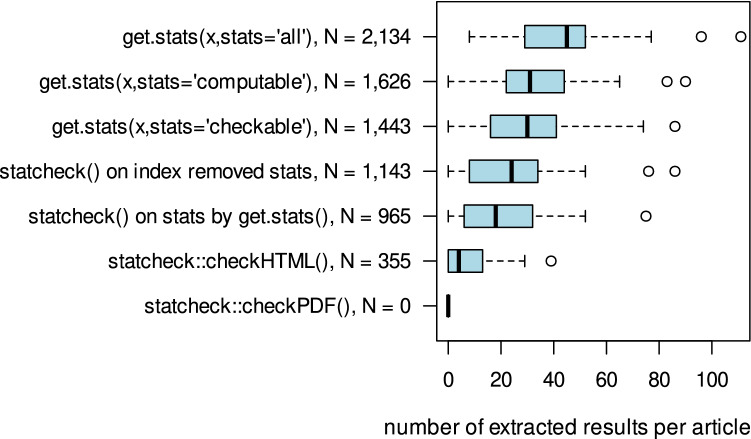
Total sums of extracted test results per method and distributions of number of extracted results per article and method.

Figure 2 displays the distribution of all detected statistical standard results per paper for the different extraction methods and input formats, with no restrictions to significant results nor type or text parts. No manually coded data exists for this analysis. In total, *get.stats()* identifies 2,134 statistical standard results in the abstracts and full text parts. 1,626 of these results are reported in a manner, that enables a recomputation of *p* values. 1,443 results are checkable. The preprocessed and further index removed vector extracted by *get.stats(x,output="stats")* increases statchecks detection rate from 355 to 965, or even 1,143 results respectively. No false positive inclusion of a checkable result by *get.stats()* was observed.

### Analysis of a large article collection with varying publishers and input formats

Next, the collection of all published PDF files by 10 mayor journals of psychology as well as all ever published XML documents by 2 open access journals is used to extend the evaluation of *get.stats()* to a bigger data set. The absolute and relative frequency of documents with extractable results per journal, different algorithm settings and input formats is listed in Table 3.

**Table 3:** Absolute and relative frequency of articles with extractable, computable or checkable statistic by journal, input format, additive settings and algorithm.

Package:		**JATSdecoder**						**statcheck**		
Function:		*get.stats(x,output=c(‘stats’,standardStats))*						*statcheck()*	*checkHTML()*	*checkPDF()*
Input format:		CERMXML/native XML*						pre processed text	CERMXML/ XML*	PDF
Extra arguments: ‘*T2t*’ and ‘*R2r*’				TRUE	TRUE	TRUE	TRUE			
Extra argument: ‘*estimateZ*’					TRUE		TRUE			
Extracted results:		‘all stats’	‘standardStats’	‘comp.’	‘comp.’	‘check.’	‘check.’	‘check.’	‘check.’	‘check.’
Journal	N articles	Absolute and relative frequency of articles with extractable results								
Behavioral Neuroscience	783	713 (91%)	706 (90%)	643 (82%)	643 (82%)	633 (81%)	633 (81%)	616 (79%)	0 (0%)	0 (0%)
Depression & Anxiety	1261	1183 (94%)	1058 (84%)	529 (42%)	547 (43%)	413 (33%)	429 (34%)	328 (26%)	267 (21%)	277 (22%)
J. of Abnormal Psychology	966	926 (96%)	899 (93%)	629 (65%)	647 (67%)	610 (63%)	625 (65%)	589 (61%)	0 (0%)	0 (0%)
J. of Child Psych. & Psychiatry	1497	1295 (87%)	1155 (77%)	681 (45%)	708 (47%)	661 (44%)	687 (46%)	560 (37%)	543 (36%)	563 (38%)
J. of Family Psychology	1146	1131 (99%)	1,102 (96%)	797 (70%)	836 (73%)	773 (67%)	810 (71%)	733 (64%)	1 (0%)	7 (1%)
J. of Management	839	638 (76%)	559 (67%)	271 (32%)	275 (33%)	231 (28%)	236 (28%)	157 (19%)	169 (20%)	171 (20%)
Pers. and Social Psychology Bul.	1341	1332 (99%)	1330 (99%)	1219 (91%)	1237 (92%)	1204 (90%)	1218 (91%)	1169 (87%)	1179 (88%)	1182 (88%)
Psychological Medicine	2924	2741 (94%)	2542 (87%)	1162 (40%)	1208 (41%)	1086 (37%)	1129 (39%)	818 (28%)	540 (18%)	579 (20%)
Psychology & Aging	1031	1022 (99%)	1006 (98%)	789 (77%)	812 (79%)	776 (75%)	795 (77%)	729 (71%)	0 (0%)	0 (0%)
Psychophysiology	1743	1708 (98%)	1671 (96%)	1461 (84%)	1473 (85%)	1439 (83%)	1448 (83%)	1361 (78%)	592 (34%)	696 (40%)
Sum in CERMXML/native PDF	13,531	12,689 (93.8%)	12,028 (88.9%)	8181 (60.5%)	8386 (62%)	7826 (57.8%)	8010 (59.2%)	7060 (52.2%)	3291 (24.3%)	3475 (25.7%)
Frontiers in Psychology	18,744*	14,362 (77%)*	13,091 (70%)*	9222 (49%)*	9408 (50%)*	8902 (47%)*	9070 (48%)*	7734 (41%)*	4465 (24%)*	
PLoS One	23,730*	22,675 (96%)*	20,211 (85%)*	8432 (36%)*	8558 (36%)*	8043 (34%)*	8153 (34%)*	6573 (28%)*	2815 (12%)*	
Sum in native XML files	42,474	37,037 (87.2%)	33,302 (78.4%)	17,654 (41.6%)	17,966 (42.3%)	16,945 (39.9%)	17,223 (40.5%)	14,307 (33.7%)	7280 (17.1%)	
Total Sum	56,005	49,726 (89%)	45,330 (81%)	25,835 (46%)	26,352 (47%)	24,771 (44%)	25,233 (45%)	21,367 (38%)	10,571 (19%)	3475 (26%)

In 89% of all processed documents *get.stats()* extracted at least one statistical result (operator between letter-number combination and number). In 46% of all analyzed articles *get.stats()* detects at least one computable result and in 44% at least one checkable result (both with arguments ‘T2t’ and ‘R2r’ set to TRUE). Activating *get.stats()*’s argument ‘estimateZ’ has a small effect (+1%) on the total sum of identified documents with computable and checkable results.

In every journal and input format, all *statcheck* functions detect fewer documents with checkable results. In 38% of all articles *statcheck()* finds checkable results within the extracted sticked results by *get.stats()*, *checkHTML()* in 19% of all CERMXML/XML files and *checkPDF()* in 26% of all PDF files. All or most articles by four journals cannot be handled by *statcheck*’s functions *checkHTML()* and *checkPDF()*, as the compiled PDF files contain incorrectly converted operators.

The amount of articles that contain computable and/or checkable results varies greatly between journals. Overall the journal *Personality and Social Psychology Bulletin* contains checkable results in 91% of the articles, compared to 34% of all articles distributed by *Depression and Anxiety*.

The preprocessed text vector that is returned by *get.stats(x,output=‘stats’)* enhances *statcheck()*’s ability to detect documents with checkable results in every journal. Both format specific *statcheck* functions *checkHTML()* and *checkPDF()* identify less documents in every journal.

**Table 4:** Total sum of extractable, computable and checkable statistics by journal, input format, additive settings and algorithm.

Package:		**JATSdecoder**						**statcheck**		
Function:		get.stats(x,output=c(‘stats’,standardStats))						*statcheck()*	*checkHTML()*	*checkPDF()*
Input format:		CERMXML						processed text	CERMXML	PDF
Extra arguments: ‘*T2t*’ and ‘*R2r*’				TRUE	TRUE	TRUE	TRUE			
Extra argument: ‘*estimateZ*’					TRUE		TRUE			
Extracted statistics		‘all stats’	‘standardStats’	‘comp.’	‘comp.’	‘check.’	‘check.’	‘check.’	‘check.’	‘check.’
Journal	N articles	total number of extracted results								
Behavioral Neuroscience	783	26,239	22,274	14,365	14,370	13,517	13,522	12,658	0	0
Depression & Anxiety	1261	29,930	15,091	4376	4615	3319	3512	2518	2095	2359
J. of Abnormal Psychology	966	33,093	22,893	8902	9255	8372	8669	7922	0	0
J. of Child Psych. & Psychiatry	1497	38,093	20,244	6686	7243	6195	6667	5093	4969	5390
J. of Family Psychology	1146	32,064	20,343	5642	6627	5137	5944	4707	1	11
J. of Management	839	16,247	10,210	2028	2246	1544	1726	998	1013	1063
Pers. and Social Psychology Bul.	1341	89,066	53,733	28,377	31,410	27,229	29,588	25,159	26,221	26,261
Psychological Medicine	2924	69,799	41,633	10,415	10,858	9452	9821	7373	3922	4579
Psychology & Aging	1031	44,318	30,653	16,314	17,071	14,993	15,506	12,751	0	0
Psychophysiology	1743	68,170	49,853	30,415	30,941	28,799	29,115	25,436	10,245	13,143
Sum in CERMXML/native PDF	13,531	447,019	286,927	127,520	134,636	118,557	124,070	104,615	48,466	52,806
Frontiers in Psychology	18,744	458,136	287,485	127,036	132,771	120,675	125,032	98,842	37,555	
PLoS One	23,730	663,400	407,117	115,873	118,765	107,959	110,338	85,463	26,915	
Sum in native XML files	42,474	1,121,536	694,602	242,909	251,536	228,634	235,370	184,305	64,470	
Total sum	56,005	1,568,555	981,529	370,429	386,172	347,191	359,440	288,920	112,936	52,806
Mean		31.5	21.7	14.3	14.7	14	14.2	13.5	10.7	15.2
SD		26.1	19.9	14.8	15	14.4	14.6	13.8	12	15.5
median		25	16	10	10	9	10	9	7	10
IQR		[14; 42]	[8; 29]	[4; 20]	[4; 20]	[4; 19]	[4; 19]	[4; 18]	[3; 14]	[4; 22]
Quantile99		125	93	69	70	68	68	65	56.3	71
Max		406	329	184	199	184	199	149	126	126
Total time in seconds		1172	463	415	416	419	424	563	153	70
Seconds per paper per processor		1.256	0.496	0.445	0.446	0.449	0.455	0.603	0.68	0.311

Non checkable results reported with ‘ns’ are removed from statchecks output, descriptive measures are calculated on those articles with detected results per setting.

Table 4 lists the total number of extracted results, standard results, as well as computable and checkable results in each setting and gives descriptive measures for those articles that contain extractable results. In total, *get.stats()* extracts 1,568,555 sticked results, 981,529 statistical standard results out of which 386,172 represent computable and 359,440 checkable results. Compared to the *statcheck* algorithms, the total sum of detected checkable results by *study.character()* is higher in every journal and input format. 12,249 computable results become checkable when activating *get.stats()*’s option to compute *p* values on estimated Z-values (from 347,191 to 359,440).

Within those articles that contain checkable results, the mean number of detected results is 14.2 with *get.stats()* and 13.5 with *statheck()* on the same preprocessed result vector, 10.7 with *checkHTML()* but 15.2 with *checkPDF()*. Also, the median, interquartile range (IQR), .99 quantile and maximum of checkable results detected by *get.stats()* are higher than *statcheck()*’s measures when processing the same vector and relevantly higher to *checkHTML()* and *checkPDF()*. *get.stats()* detects the highest number of checkable results in one study with 199 results, whereas *statcheck()* identifies 149 results as maximum.

No unexpected processing times occurred. As many preprocessing operations are performed, the extraction of the sticked results with *get.stats(x,output=‘stats’)* takes 1.3 seconds on average per paper and processor. The mean processing time of this vector differs slightly between *statcheck()* (.6 sec.) and *get.stats()* (.5 sec. per document and processor). In total, both file specific *statcheck* functions work a lot faster, as no case specific letter conversion nor uniformization is performed before extracting the results.

**Table 5:** Increase factor of detection rate for checkable results by get.stats() compared to statcheck’s functions.

Journal	get.stats() versus *statcheck(get.stats(x,* *output="stats"))*	get.stats() versus *checkHTML()*	get.stats() versus *checkPDF()*
Behavioral Neuroscience	1.07	Inf	Inf
Depression & Anxiety	1.39	1.68	1.49
J. of Abnormal Psychology	1.09	Inf	Inf
J. of Child Psychology & Psychiatry	1.31	1.34	1.24
J. of Family Psychology	1.26	5944.00	540.36
J. of Management	1.73	1.70	1.62
Personality and Social Psychology Bul.	1.18	1.13	1.13
Psychological Medicine	1.33	2.50	2.14
Psychology & Aging	1.22	Inf	Inf
Psychophysiology	1.14	2.84	2.22
Frontiers in Psychology	1.26	3.33	
PLoS One	1.29	4.10	

Table 5 displays the increase factors with which *get.stats()* identifies more checkable results per journal. As no PDF files are analyzed for *Frontiers in Psychology* and *PLoS One*, these fields are left blank for *checkPDF()*. *get.stats()* outperforms *statcheck()* in detecting checkable results by a varying factor of 1.07 for *Behavioral Neuroscience* to 1.73 for *Journal of Management* when processing the same preprocessed vector of sticked results extracted with *get.stats(x,output="stats")*. This pattern holds for *checkHTML()* when processing *CERMINE* converted PDFs (1.13 to 2.84) and *checkPDF()* processing the original PDF files. Three PDF article sets mostly contain non-standard coded operators and cannot be processed in their native version by *checkPDF()* nor in their *CERMINE* compiled version by *checkHTML()*. Compared to *checkHTML()* *get.stats()* extracts 3.33 (*Frontiers in Psychology*) to 4.1 (*PLoS One*) times more checkable standard results within the native XML files with most results coded in HTML style.

## Conclusion

*get.stats()*’s high precision and flexibility in extracting statistical results from research papers in NISO-JATS formatted XML files has been demonstrated. It facilitates plausibility checks on many standard results reported in text, and can help scientists as well as editors to summarize and check a study regarding reporting style and checkability of reported results. However, fully reported and plausible results do not tell us anything about the methodological quality of a study.

*get.stats()* is heavily outperforming all three *statcheck*’s algorithms in extracting statistics from floating text. The vague definition of a statistical result being any letter-number combination pointing to a number with an operator makes *get.stats()* a very general and valid tool to detect statistical results within text. Incomputable, computable and checkable results become clearly distinguishable. If possible, *p* values are recomputed and become checkable if also reported. Incompletely or inconsistently reported results can be detected by a manual check of reported and computed *p* values.

*JATSdecoder*’s functions can handle most PDF and *CERMINE* specific conversion errors in statistical results, except in cases with non compiled text parts (e.g. footnotes, listings, section titles). Incorrectly converted operators and some Greek letters are corrected, while completely missing operators are replaced with ‘$<=>$’ for many statistical results. The extracted vector of sticked results by *get.stats()*, converts CERMINE converted PDF files, that are unprocessable for *checkPDF()*, into a format that is post-processable with *statcheck()*.

The results of Nuijten et al.^20^ could not be replicated with neither input format. Compared to the original paper, *checkPDF()* does not detect a single checkable result in the PDF files, while *checkHTML()* just detects a small proportion in the browser generated HTML files. Finally, *statcheck()* identifies more checkable results within the preprocessed output of *get.stats()* than were found by Nuijten et al.^20^. Therefore, *get.stats()* preprocessed output enhances any automated plausibility check with *statcheck()*, especially for those PDF files that compile with errors, which applies to full article collections of some journals.

In all cases, *get.stats()* outperforms all *statcheck* algorithms. Even compared to a manual extraction, its precision on extracting statistical results from text can be considered very high. In some rare cases, the compilation by *CERMINE* failed to cover all text parts, leading to some undetected results. However, this problem only needs to be considered when PDF conversion was applied.

Most deviations observed to the manually coded data by Wicherts et al.^27^ are caused by their representation in tables, differing inclusion criteria and/or differing definitions of a checkable result. No false positive detections of checkable results by *get.stats()* were observed.

A non-negligible part of all reported results in the surveyed articles is presented in tables and cannot be extracted nor checked by neither *get.stats()* nor *statcheck*. Converting tables in PDF files to text mostly produces spurious artifacts in the resulting output, as they allow very individual layout and coding styles. *statcheck* detects results reported in tables if they are reported in a full textual manner in one cell of the table, which is a rather rare event. Up to now, as only a very small portion of tabulated results can be extracted with *statcheck*, it is sensible to restrict checking procedures to results reported within the main text only. Descriptive measures of the total number of reported results in text therefore tend to be mostly negatively biased estimates for the actual number of reported results. Correlation matrices and regression tables often contain a high amount of test results. For test results reported with asterisks instead of *p* values, a precise plausibility check is generally not possible.

As no algorithm can be perfect, false positive and negative detections may occur when *get.stats()* tags a reported result as a standard result. Many PDFs lose their special characters during conversion to NISO-JATS coded XML files which may lead to false positives and negatives, when a missing Greek letter other than $\chi$ is used but $\chi$ is imputed by *letter.convert()*. Results that are labeled equally to the above defined standard results but represent other measures, will be treated as a standard result. Especially wrongly interpreted Z-values (e.g. in a coordinate: ‘x = 1, y = 2, z = 3’) will automatically lead to the computation of a *p* value and suggest that the result is computable. Special or anomalous labels of results and special letter uses that are not captured by *get.stats()* may lead to a non-detection as checkable standard result.

*JATSdecoder* enables a wide range of possibilities for meta-analytical research and mirroring techniques. The reported degrees of freedom in some test statistics allow an estimation of the sample size which a study is based on. Another option is to analyze all ever reported statistics by an author, affiliation, subject and/or other subsets of metadata. A p-curve analysis of the reliably extracted results from one or many article/s may help to identify questionable research practices performed by individuals or groups. Its ability to split an article into selectable sections and phrases enables sentence detection in specific text parts of a study (e.g. discussion/conclusion only). With a little additive text extraction effort, it is possible to detect all investigated variables or effects within a research topic.

## Data Availability

*JATSdecoder* software is freely available at: https://github.com/ingmarboeschen/JATSdecoder A simple web interface enables the use of *JATSdecoder*’s function *get.stats()* on single files of different formats: www.get-stats.app Scripts to reproduce this and other analyses performed with *JATSdecoder*, as well the extracted results from *Frontiers in Psychology* and the selection of *PLoS One* articles are stored at:https://github.com/ingmarboeschen/JATSdecoderEvaluation.
